# Apigenin Alleviates Intervertebral Disc Degeneration *via* Restoring Autophagy Flux in Nucleus Pulposus Cells

**DOI:** 10.3389/fcell.2021.787278

**Published:** 2022-01-14

**Authors:** Chenglong Xie, Yifeng Shi, Zuoxi Chen, Xin Zhou, Peng Luo, Chenxuan Hong, Naifeng Tian, Yaosen Wu, Yifei Zhou, Yan Lin, Haicheng Dou, Aimin Wu, Qishan Huang, Xiaolei Zhang, Xiangyang Wang

**Affiliations:** ^1^ Department of Orthopaedics, The Second Affiliated Hospital and Yuying Children’s Hospital of Wenzhou Medical University, Wenzhou, China; ^2^ Key Laboratory of Orthopaedics of Zhejiang Province, Wenzhou, China; ^3^ The Second School of Medicine, Wenzhou Medical University, Wenzhou, China; ^4^ Department of Orthopaedics, Wenzhou Hospital of Integrated Traditional Chinese and Western Medicine, Wenzhou, China; ^5^ Chinese Orthopaedic Regenerative Medicine Society, Hangzhou, China

**Keywords:** intervertebral disc degeneration, autophagy, autophagic flux, apoptosis, senescence, TFEB

## Abstract

Oxidative stress–induced apoptosis and senescence of nucleus pulposus (NP) cells play a crucial role in the progression of intervertebral disc degeneration (IVDD). Accumulation of studies has shown that activated autophagy and enhanced autophagic flux can alleviate IVDD. In this study, we explored the effects of apigenin on IVDD *in vitro* and *in vivo*. Apigenin was found to inhibit tert-butyl hydroperoxide (TBHP)–induced apoptosis, senescence, and ECM degradation in NP cells. In addition, apigenin treatment can restore the autophagic flux blockage caused by TBHP. Mechanistically, we found that TBHP may induce autophagosome and lysosome fusion interruption and lysosomal dysfunction, while apigenin alleviates these phenomena by promoting the nuclear translocation of TFEB *via* the AMPK/mTOR signaling pathway. Furthermore, apigenin also exerts a protective effect against the progression of IVDD in the puncture-induced rat model. Taken together, these findings indicate that apigenin protects NP cells against TBHP-induced apoptosis, senescence, and ECM degradation *via* restoration of autophagic flux *in vitro*, and it also ameliorates IVDD progression in rats *in vivo*, demonstrating its potential for serving as an effective therapeutic agent for IVDD.

## Introduction

Intervertebral disc degeneration (IVDD) is a worldwide age-related musculoskeletal disease, resulting in low quality of life and an increase in the clinical and socioeconomic burdens (Vergroesen et al., 2015). It has been reported that over 40% of low back pain is due to the IVDD progress (Clark and Horton, 2018), which is a consequence of multiple pathological factors, such as abnormal stress, aging, genetic predisposition, and obesity (Kanayama et al., 2009; Ikegawa, 2013). Although there are many studies targeting the IVDD progress, the exact underlying pathological mechanism of IVDD is still unclear. The intervertebral disc (IVD) is composed of three integrated structures: a jelly-like tissue, nucleus pulposus (NP); multiple layers of collagen fibers, annulus fibrosus (AF); and the cartilaginous endplates in the upper and lower vertebra. The NP cells, mainly regulating the extracellular matrix metabolism (ECM) and controlling the balance of proteoglycan and type II collagen production (Ashley et al., 2016), are the most critical components of the disc to maintain the disc function and flexibility (Banala et al., 2018). Although IVDD is associated with multiple pathological processes, it is characterized by decreased intervertebral space height and dysfunction of NP and AF. Besides, previous studies indicated that the production of reactive oxygen species (ROS) induced by oxidative stress may increase the level of apoptosis and senescence in NP cells, which may directly affect the ECM anabolism and catabolism (Olga et al., 2016; Feng et al., 2017; Yu et al., 2018). Therefore, targeting oxidative stress–induced apoptosis and senescence in NP cells is regarded as an effective therapeutic strategy for alleviating IVDD progress.

Macroautophagy (autophagy), a well-known highly conserved substantial intracellular degradation process, exhibits its cytoprotective effect by scavenging the unwanted senescent organelles and misfolded proteins (Zheng et al., 2019b; Deretic, 2021). Autophagy is a dynamic process consisting of several steps: subcellular membranes undergo structural changes, then autophagosomes come into being and fuse with lysosomes, and finally the fused autophagolysosomes degrade the content and release the energy (Nakatogawa, 2020). The entire dynamic process is described as “autophagic flux,” which can be used to assess autophagic activity. Many research studies demonstrated that the autophagic flux was closely related to cell senescence and apoptosis in IVDD development (Zheng et al., 2019a; Hu et al., 2021). As previously reported, autophagic flux blockage existed in NP cells under oxidative stress (Liu et al., 2020). In the meantime, the oxidative stress or inflammatory factors, including TBHP and interleukin-1, mediated ECM degradation which would worsen after chloroquine (CQ) administration, a classical autophagic flux blocker (Xu et al., 2015; Chen et al., 2018). Hence, enhancing autophagy and restoring autophagic flux might also serve as a potential therapeutic strategy for IVDD.

Apigenin is a natural flavonoid that exists in multiple plants, vegetables, fruits, spices, and Chinese herbal medicines (Shankar et al., 2017; Sharma et al., 2019). A bunch of research studies indicated that apigenin exerts anti-inflammatory (Wang and Huang, 2013), antioxidant (Yang et al., 2020), anticancer (Guedj et al., 2020), and antiplatelet aggregation effect (Sharifi-Rad et al., 2021) in various diseases, such as neurodegenerative diseases and epilepsy, which can alleviate pathogenesis and symptoms of them (Sang et al., 2020; Sharma et al., 2020). In recent studies, apigenin showed the ability to induce autophagy and improve autophagic flux in various tissues, such as human skin, cartilage, heart, and retina (Bridgeman et al., 2016; Cho et al., 2019; Zhang et al., 2020b). However, whether the protective effects of apigenin exist in NP cells is still unclear. Therefore, we investigated the effects of apigenin *in vitro* and *in vivo* and explored the underlying mechanism.

## Materials and Methods

### Ethical Statement

All surgical operations and treatments on animals were approved by the Animal Care and Use Committee of Wenzhou Medical University.

### Reagents and Antibodies

Apigenin (C15H10O5; purity ≥98%), hematoxylin–eosin (HE) staining kit, and Safranin O-fast green (SO) staining kit were purchased from Beijing Solarbio Science & Technology (Beijing, China); tert-butyl hydroperoxide solution (TBHP), chloroquine (CQ), and type II collagenase were obtained from Sigma-Aldrich (St. Louis, MO, United States); dorsomorphin (compound C) and bafilomycin A1 were purchased from MCE (Monmouth Junction, NJ, United States); the primary antibodies against Bcl2 (12789-1-AP), Bax (50599-2-Ig), MMP13 (18165-1-AP), β-actin (20536-1-AP), and TFEB (13372-1-AP) were obtained from Proteintech (Wuhan, China); p21 (ab109199), p62 (ab109012), collagen II (ab188570), ADAMTS5 (ab41037), AMPK (ab32047), p-AMPK (ab13448), LAMP2 (ab125068), and lamin B (ab133741) were acquired from Abcam; LC3 (#83506), cleaved caspase 3 (#9661), p-mTOR (#5536), and mTOR (#2972) and apigenin were partially purchased from Cell Signaling Technology (Danvers, MA, United States); p16INK4a (A0262), aggrecan (A11691), and LAMP1 (A2582) were purchased from ABclonal Technology (Wuhan, China). Goat anti-rabbit and anti-mouse IgG-HRP antibodies were purchased from Bioworld (Nanjing, China). Alexa Fluor® 594- and 488-conjugated secondary antibodies were obtained from Jackson ImmunoResearch (PA, United States); 4′,6-diamidino-2-phenylindole (DAPI) was purchased from Beyotime (Shanghai, China).

### Rat NP Cells Culture and Viability Analysis

Healthy male Sprague**–**Dawley (100–150 g, 4 weeks) were used for NP cell extraction. The methods for NP cell extraction and culture were described in a previous study (Xie et al., 2021).

Cell viability analysis was performed by CCK-8. NP cells were seeded in 96-well plates and incubated with different concentrations of apigenin or TBHP alone, or with both of them. Then, to each well was added 100 μl solution that had a combination of 90 μl DMEM/F12 and 10 μl CCK-8 solution, and incubated for 120 min at 37°C; after that, the absorbance was measured using the microplate reader at 450 nm.

Live and dead NP cells seeded in 6-well plates were also detected after 24-h incubation with TBHP or API via the Calcein/PI Cell Viability/Cytotoxicity Assay Kit according to the manufacturer’s instructions. The cells were observed and scanned by using a microscope (Olympus Inc., Tokyo, Japan).

### Western Blotting

The total cytoplasmic and nucleus proteins were extracted from NP cells after various treatments or stimulation *via* related kits according to the manufacturer’s instructions (Beyotime, China). Then the balanced protein samples were separated *via* sodium dodecyl sulfate–polyacrylamide gel electrophoresis (SDS-PAGE) and transferred to a polyvinylidene difluoride membrane (PVDF) (Bio-Rad, United States), the obtained membranes were blocked by 5% (w/v) non-fat milk for 120 min and then incubated with the primary antibody, whose concentration ranges from 1:500 to 1:1,000, overnight at 4°C, and subsequently, incubated with the corresponding secondary antibody for 120 min at room temperature. A ChemiDoc XRS + Imaging System (Bio-Rad) was used for visualizing the membranes, and Image Lab 3.0 software (Bio-Rad) was for quantification.

### Small-Interfering RNA Transfection

The specific rat TFEB small-interfering RNA (siRNA) was obtained from GenePharma (Shanghai, China), and negative control or siRNA-TFEB were transfected into NP cells using Lipofectamine 2000 siRNA transfection reagent (Thermo Fisher, UT, United States) according to the manufacturer’s instructions.

### SA-β-Gal Staining

Senescence activity of NP cells was assessed *via* SA-β-gel staining kit (Beyotime), according to the instructions. After staining, the cells turned blue, indicating that cells showed higher SA-β activity. And the degree of senescence activity was presented by the percentage of staining positive cells to total cells in each filed of slides.

### TUNEL Staining

TUNEL staining, targeting at detecting cell apoptotic DNA fragmentations, was used for assessing the apoptotic level of NP cells. Normal or treated NP cells were fixed in the slides, then incubated and stained with corresponding reagents for 30 min at room temperature according to the manufacturer’s instructions (F. Hoffmann-LaRoche Ltd., Basel, Switzerland), and finally stained with DAPI for visualizing the nuclei. The obtained slides were visualized and captured by using a fluorescence microscope (Olympus Inc., Tokyo, Japan).

### Lyso-Tracker Red Staining

The function of lysosomes was assessed *via* Lyso-Tracker Red (LTR) staining; 50 nM LTR was chosen for staining with NP cells (Invitrogen, Grand Island, NY) for 30 min at 37°C and then Hoechst 33258 was added to stain nuclei for 10 min. After that, bright and strong fluorescent staining of lysosomes can be observed under a fluorescence microscope (Olympus Inc., Tokyo, Japan), and fluorescence intensity was analyzed by ImageJ (Bethesda, MD, United States).

### Immunofluorescence

As described previously (Xie et al., 2021), slides were incubated with primary antibodies, collagen II (1:100), MMP-13 (1:200), LC3 (1:100), and LAMP1 (1:200), overnight at 4°C, and then labeled with suitable secondary antibodies for 60 min at 37°C. The nuclei were stained with DAPI for 5 min. All the images were observed and captured by using a fluorescence microscope (Olympus Inc., Tokyo, Japan).

### Puncture-Induced Rat Intervertebral Disc Degeneration Model

The animal experimental protocols were approved by the Animal Care and Use Committee of Wenzhou Medical University. Male Sprague–Dawley rats (8 weeks, 200–250 g, *n* = 24) were obtained from the Animal Center of the Chinese Academy of Sciences (Shanghai, China). Animals were bred in a suitable condition with a 12-h light/dark cycle and were regularly fed. Sprague-Dawley rats were randomly divided into three groups: sham group, IVDD group, and IVDD + apigenin group. After being anesthetized by an intraperitoneal injection of 2% (w/v) pentobarbital (40 mg/kg), a 26 g needle was punctured into the disc as previously described (Xie et al., 2021). Once the IVDD model was accomplished, except the regular feeding, the sham group were given saline every day *via* intragastric administration as well as the IVDD group; in the meantime, the IVDD + apigenin group was given 10 mg/kg apigenin (dissolved in saline) once a day for 8 weeks until killed. The rats were given daily care to ensure their survival.

### X-Ray Image Acquisition and Magnetic Resonance Imaging

X-ray and MRI were taken 8 weeks after surgery. X-ray of rat tails was captured by an imaging machine (Kubtec, United States) in a prone position. Similarly, MRI imaging was performed on rat tails through a 3.0 T clinical magnet (Philips Intera Achieva 3.0 MR) (Zheng et al., 2019a). The Pfirrmann grading system was used for assessing IVDD degree (Pfirrmann et al., 2001).

### Histopathological Analysis and Immunohistochemical Examination

Surgery and non-surgery rat tails were harvested after being killed by an overdose of anesthesia at 8 weeks, respectively. The obtained specimens were sequentially decalcified, fixed, dehydrated, and eventually embedded in paraffin. Then the tissues were cut into 5-μm sections. HE staining and SO staining were performed on 5-μm-thick sectioned tissues that target at assessing the cellularity and morphology variation of the intervertebral disc under a microscope (Olympus Inc., Tokyo, Japan). As for immunohistochemical examination, the sliced tissues underwent deparaffinization and dehydration, then pepsin incubation for 30 min, followed by 1% (w/v) goat serum albumin block for 60 min at room temperature, and then primary antibody (1:200) incubation at 4°C overnight. Finally, tissues were incubated with appropriate HRP-conjugated secondary antibodies. Images were observed by Image-Pro Plus software, version 6.0 (Media Cybernetics, Rockville, MD, United States).

### Statistical Analysis

Data were expressed as means ± standard deviation (SD) and analyzed by GraphPad Prism 9 (United States). One-way analysis of variance (ANOVA) was performed to assess the difference among groups and Tukey’s test for comparison between groups. Two group differences were analyzed by Student’s t-test. *p* values <0.05 were considered statistically significant.

## Results

### Effects of Apigenin on Cell Viability

**FIGURE 1:**
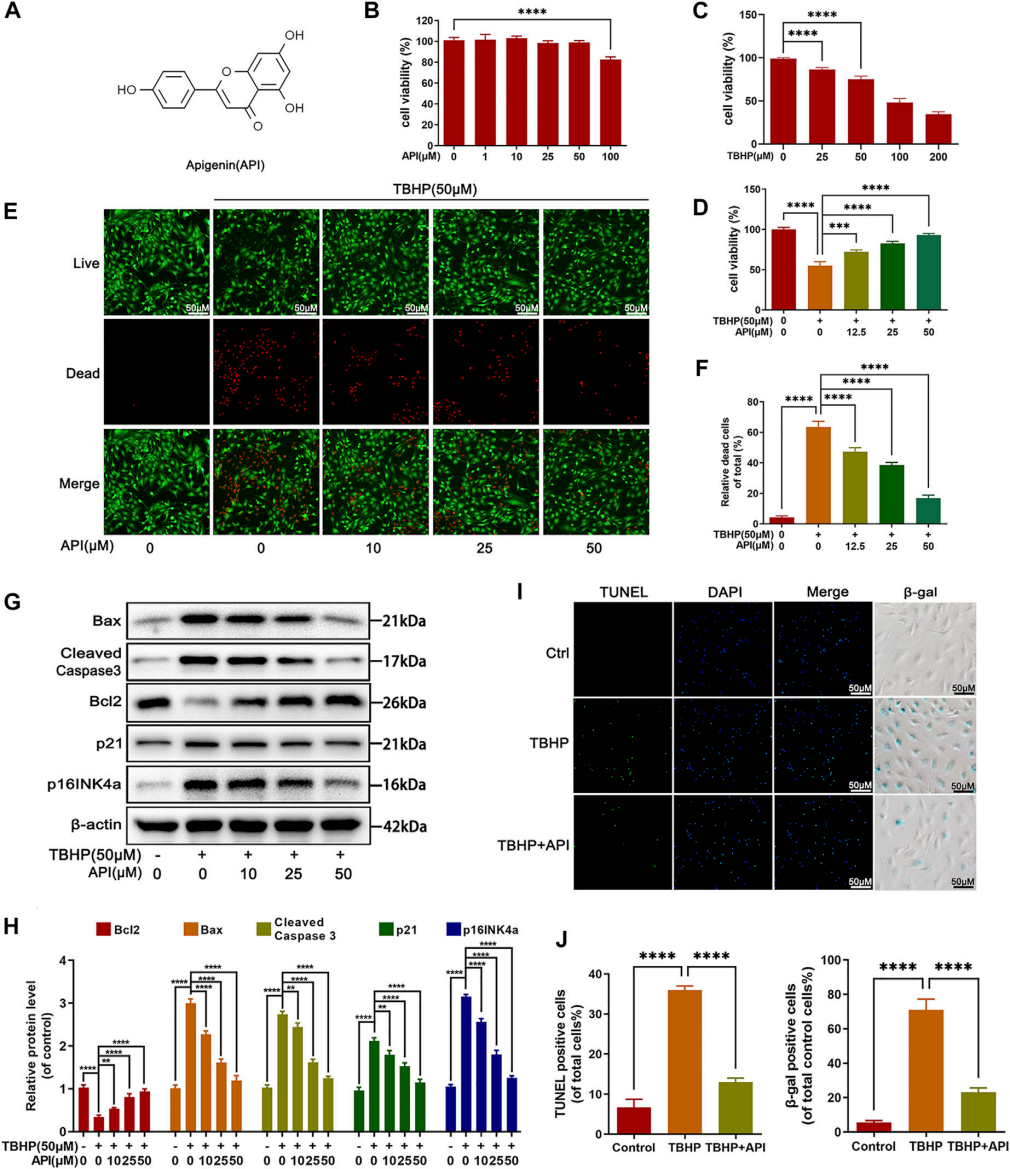
Effects of apigenin (API) on cell viability, apoptosis, and senescence of rat nucleus pulposus (NP) cells. **(A)** Chemical structure of API. **(B,C)** Counting Kit-8 (CCK8) results of NP cells treated with various concentrations of API or TBHP for 24 h. **(D)** CCK-8 results of NP cells co-treated with API and TBHP (50 μM) for 24 h. **(E,F)** Live and dead staining of NP cells cocultured with API and TBHP (50 μM) for 24 h. (scale bar: 50 μm). **(G)** Western blot results of Bax, cleaved caspase 3, Bcl2, p21, and p16INK4a expression levels in NP cells. **(H)** Quantification of Bax, cleaved caspase 3, Bcl2, p21, and p16INK4a by immunoblot. **(I)** TUNEL and SA-β-gel staining was performed in NP cells (scale bar: 50 μm). **(J)** The positive cells in TUNEL and SA-β-gel staining were quantified. The data in the figures represent mean ± S.D. Significant differences between groups are indicated as ****p < 0.0001, ***p < 0.001.

The chemical structure of apigenin is shown in Figure 1A. Toxicity analysis of apigenin was performed in this study, as observed in Figure 1B, and no cytotoxic effect was found when NP cells were incubated in various concentrations of apigenin for 24 h; and TBHP decreased the cell viability of NP cells in a dose-dependent manner, which was reversed by apigenin treatment (Figures 1C,D). Besides, live and dead cell staining also confirmed this result (Figures 1E,F).

### Apigenin Suppresses TBHP-Induced Apoptosis and Senescence in NP Cells

Several experiments were performed to investigate the effect of apigenin on apoptosis and senescence under TBHP stimulation. Senescence-related indicators, p16INK4a and p21 protein levels in NP cells and SA-β-gal activity of NP cells were assessed in this study. As shown in Figures 1F–I, both protein level of p16INK4a and p21, and the positive SA-β-gal cells were increased by TBHP, which were reversed by apigenin treatment in NP cells. Next, TUNEL staining, a classical damaged DNA test method, was used for apoptosis analysis. Besides, the expression level of pro-apoptotic protein (Bax and cleaved caspase 3) and antiapoptotic protein (Bcl-2) were detected by Western blot. It can be observed in Figures 1F,G that apigenin alleviated Bax and cleaved caspase 3 protein levels, while increasing Bcl2 protein levels. From the results of TUNEL staining (Figures 1H,I), more positive cells were detected in the TBHP group, while apigenin administration obviously abolished the TBHP-induced apoptotic cells. Taken together, these results demonstrate that apigenin exerts a protective effect against TBHP-induced apoptosis and senescence in NP cells.

### Apigenin Alleviates TBHP-Induced ECM Degradation in NP Cells

**FIGURE 2:**
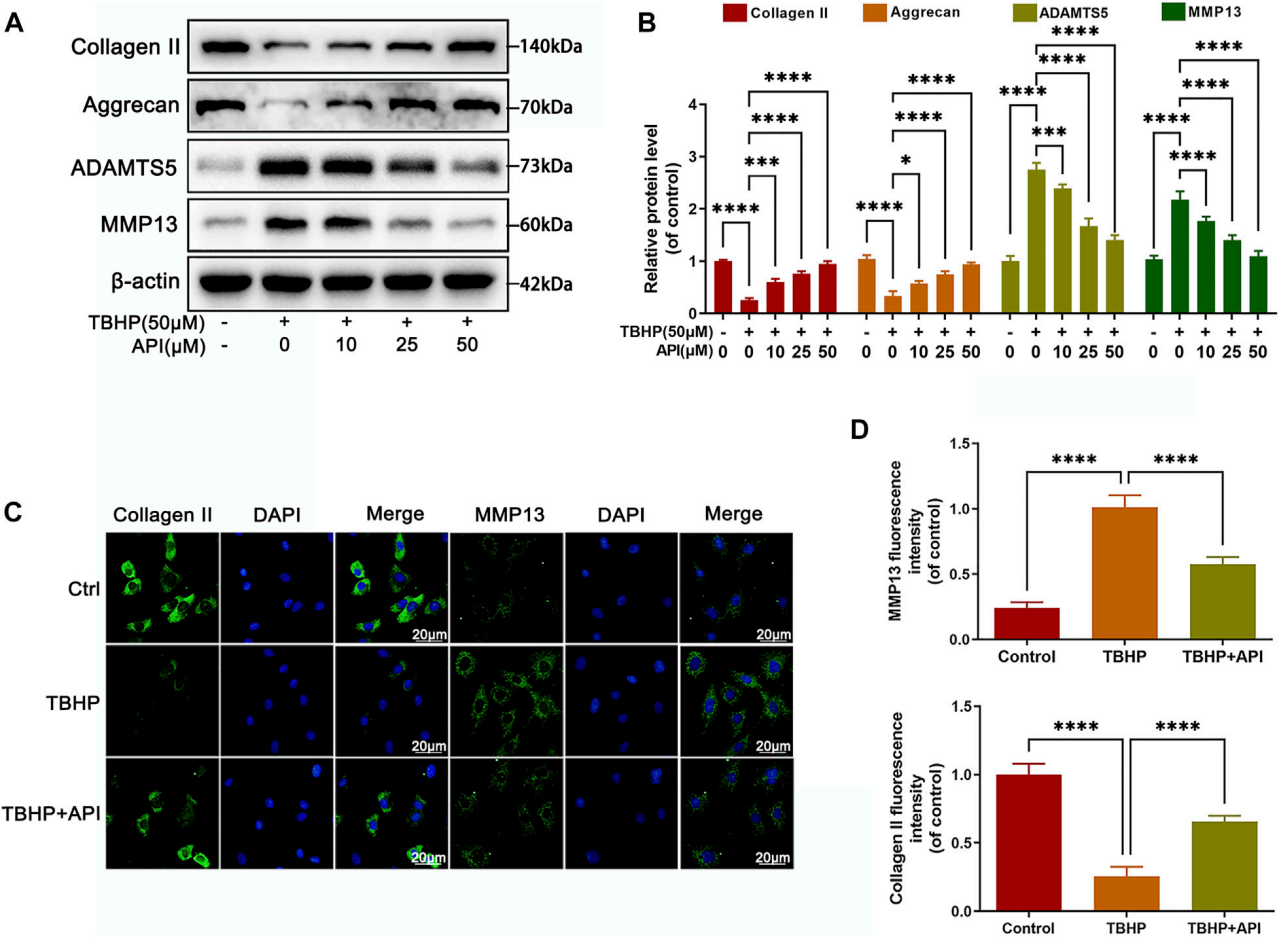
Apigenin (API) treatment alleviates TBHP-induced ECM degradation in NP cells. NP cells were treated with TBHP and various concentrations of API for 24 h. **(A)** The protein expression of collagen II, aggrecan, ADAMTS5, and MMP13 in NP cells as treated above were assessed by Western blot. **(B)** Quantification of collagen II, aggrecan, ADAMTS5, and MMP13 by immunoblot. **(C)** The cell immunofluorescence images of collagen II and MMP13 (scale bar: 20 μm). **(D)** Quantitative analysis of the fluorescence intensity of collagen II and MMP13 was done by ImageJ. Data are represented as mean ± SD. ****p < 0.0001, *p < 0.05.

Then we studied the effect of apigenin on the degradation of ECM in TBHP-treated NP cells, which was the main reason causing IVDD progress. As seen in Figures 2A,B, Western blot results proved that TBHP treatment reduced the protein expression of collagen II and aggrecan; on the contrary, it promoted the expression of matrix metalloproteinase-13 (MMP13) and a disintegrin and metalloproteinase with thrombospondin motifs 5 (ADAMTS), but apigenin partially reversed the aforemenioned results in a dose-dependent manner. In addition, the fluorescence intensity of collagen II decreased along with the increasing fluorescence intensity of MMP13 in cells exposed to TBHP (Figures 2C,D). Also, it was reversed by apigenin treatment, which was consistent with the results of Western blot. Hence, it can be concluded that apigenin protects NP cells from ECM degradation induced by TBHP stimulation by regulating the balance of ECM synthesis–related and degradation-related protein levels.

### Apigenin Promotes Autophagic Flux in NP Cells

In order to evaluate whether apigenin can activate autophagy in NP cells, several autophagy-related protein levels were analyzed through Western blot. As shown in Supplementary Figures S1A–D, the LC3-II/I protein ratio and Lamp2 expression increased, but p62 expression decreased in NP cells by apigenin treatment in a dose- and time-dependent way, respectively. Besides, the results of cell immunofluorescence staining indicated that apigenin treatment effectively enhanced the colocalization of LC3 with LAMP1 (Supplementary Figures S1E,F), which confirmed the aforementioned findings. Interestingly, LC3-II is also involved in the dynamic variation during the autophagy process; either the increased generation of autophagosomes or the decreased degeneration of autolysosomes may increase the LC3-II level. Besides, p62, a specific substrate degraded in autolysosomes, is also an indicator for the status of autophagic flux. Hence, the autophagic flux activity was assessed by LC3-II and p62 expression levels in NP cells treated with or without the bafilomycin A1 (a typical lysosomal inhibitor). As observed in Supplementary Figures S1G,H, apigenin promotes autophagic flux in NP cells. Altogether, these data indicate that apigenin activates autophagy and promotes autophagic flux in NP cells.

### Apigenin Restores Autophagy Flux Blockage Induced by TBHP Treatment in NP Cells

**FIGURE 3:**
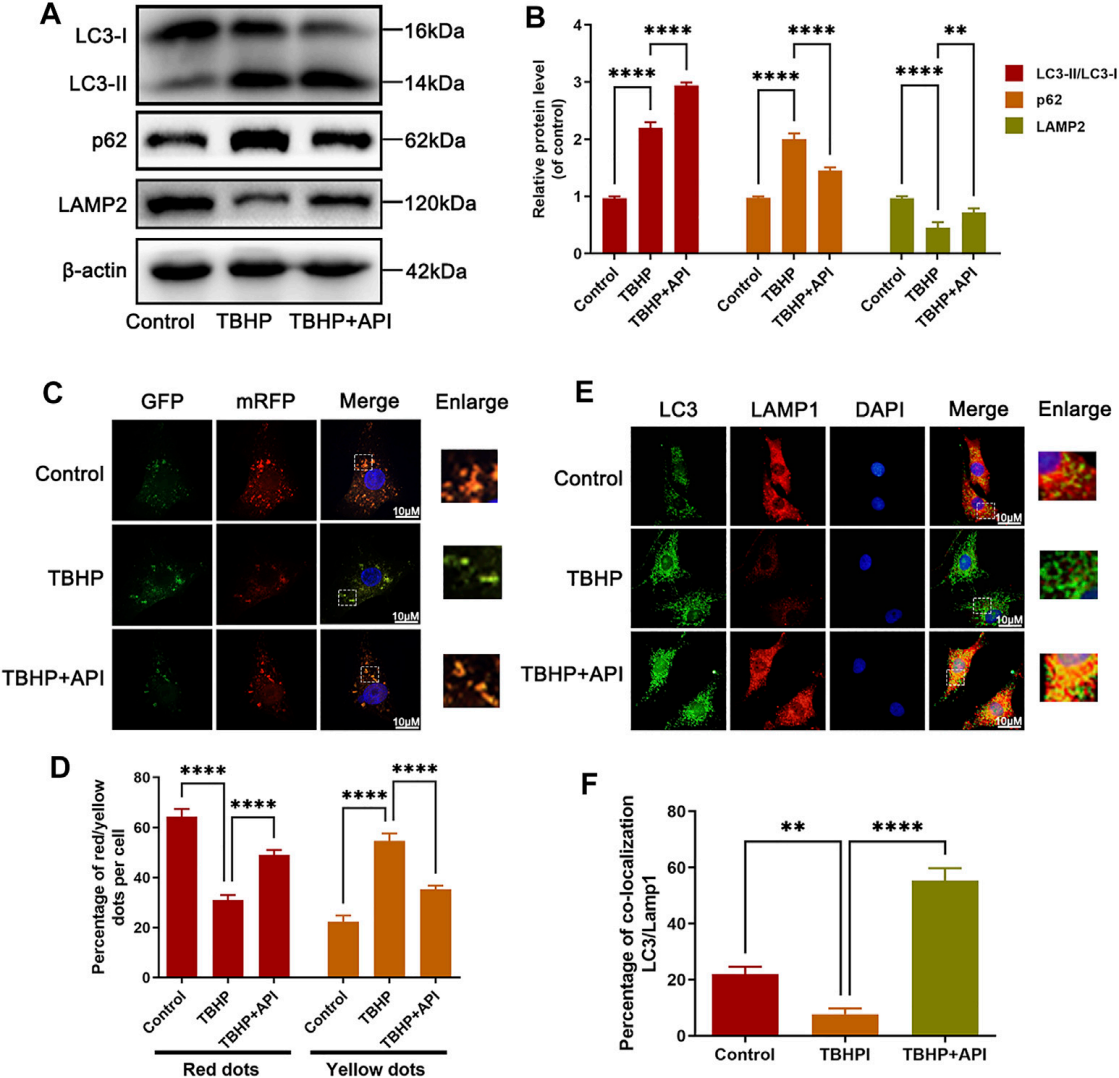
Apigenin (API) treatment restores TBHP-induced autophagic flux blockage in NP cells. NP cells were treated with API (50 μM) or TBHP (50 μM). **(A,B)** Western blot results of LC3, p62, and LAMP2 in NP cells. **(C,D)** The mRFP-GFP-LC3 staining was performed in NP cells (scale bar: 10 μm). **(E)** The immunofluorescence colocalization of LC3 and LAMP1 in NP cells (scale bar: 10 μm). **(F)** Quantification of the percentage of colocalization of LC3 and LAMP1 was analyzed by ImageJ. Data are represented as mean ± SD. ****p < 0.0001, ***p < 0.001, **p < 0.01.

The effect of apigenin on regulating autophagic flux was analyzed in NP cells exposed to TBHP. As seen from Figures 3A,B, the LC3-II/LC3-I protein ratio was elevated as well as p62 expression, indicating that NP cells exposed to TBHP caused autophagic flux blockage, while TBHP-induced upregulation of p62 expression and LC3-II/LC3-I ratio were reversed by apigenin treatment. Then, to quantize the autophagic flux, a tandem mRFP-GFP-LC3 construct was used in this study. Normally, only red fluorescence can be observed in lysosomal because of its acidic environment inhibits the green fluorescence; but yellow puncta, a merged form of red and green fluorescence, can be seen in autophagosomes. As shown in Figures 3C,D, more yellow puncta were found in the TBHP group than in the control group, indicating that autophagic flux was blocked in NP cells; but in apigenin and TBHP coculture group, fewer yellow puncta and more red fluorescence were observed compared to the TBHP group. Besides, a further analysis was performed *via* cell immunofluorescence staining. From the results in Figures 3E,F, double-immunofluorescence staining for Lamp1 (a major protein of lysosomal) and LC3 was performed, which showed that the colocalization of LC3 with Lamp1 was increased in NP cells with apigenin treatment compared with that in TBHP-treated cells; Therefore, these results demonstrate that apigenin has a positive role in restoring impaired autophagic flux in TBHP-treated NP cells.

### CQ Reverses the Protective Effects of Apigenin in NP Cells Under Oxidative Stress

To determine whether apigenin-induced autophagy and autophagic flux benefited the cell viability, we explored the effect of apigenin on apoptosis, senescence, and ECM degradation when cocultured with CQ, a well-known lysosomal cavity alkalizer that could block autophagic flux in TBHP-treated NP cells. Western blot results demonstrated that CQ treatment inhibited the apigenin’s protective function against TBHP-induced upregulation of apoptosis-related proteins (Bax and cleaved caspase 3) and senescence-related proteins (p16INK4a and p21) expression, as well as the antiapoptosis protein (Bcl2) expression level (Supplementary Figures S2A,B). Meanwhile, the percentage of TUNEL and SA-β-gal–positive cells was further confirmed the Western blot results (Supplementary Figures S2C,D). In addition, the protection of apigenin against TBHP-induced ECM degradation was also abolished by CQ, as observed from the results in Supplementary Figures S3A,B that the treatment of CQ inhibited ECM anabolic proteins (collagen II and aggrecan) levels and increased ECM catabolic proteins (MMP13 and ADAMTS5) levels. Besides, the immunofluorescence staining of collagen II and MMP13 showed the same results of Western blot (Supplementary Figures S3C–F). Taken together, the aforementioned data prove that apigenin-induced antiapoptosis, anti-senescence, and anti-degradation of ECM function were *via* autophagic flux regulation.

### Apigenin Promotes the Nuclear Translocation of TFEB in TBHP-Treated NP Cells

**FIGURE 4:**
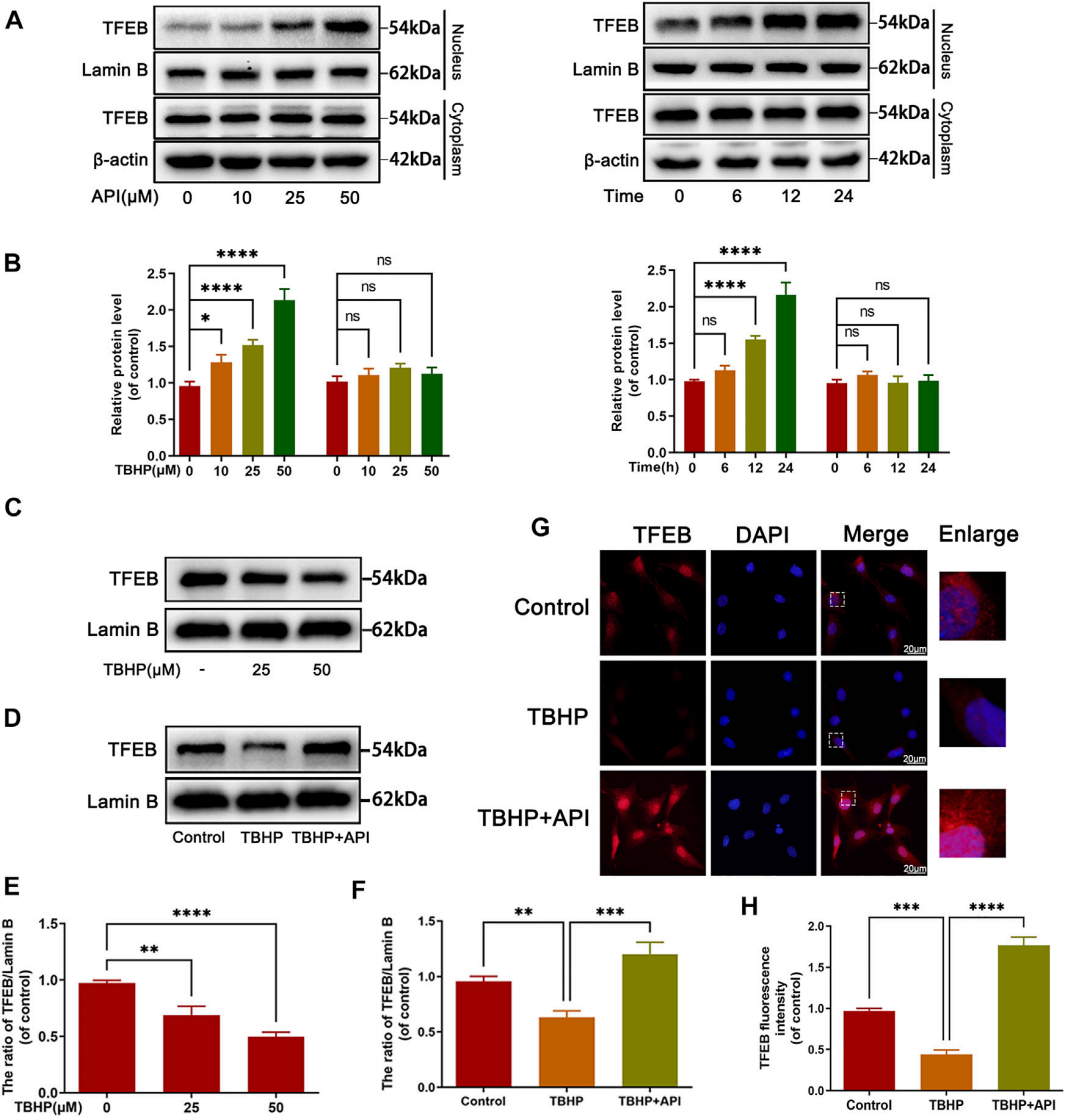
Apigenin (API) treatment promotes the translocation of TFEB to nuclear. **(A,B)** The expression of TFEB in the cytoplasm or nucleus in NP cells treated with API for different concentrations and times. **(C)** The nuclear expression level of TFEB in NP cells treated with different concentrations of TBHP. **(D)** The nuclear protein level of TFEB in NP cells treated with API (50 μM) or TBHP (50 μM). **(G)** The cell immunofluorescence of the nucleus TFEB was performed in NP cells (scale bar: 10 μm). **(E,F,H)** Quantitative analysis of TFEB by immunoblot and fluorescence intensity of TFEB was measured by software ImageJ. Data are represented as mean ± SD. ****p < 0.0001, ***p < 0.001, **p < 0.01, *p < 0.05.

Next, several experiments were performed to figure out whether apigenin regulated TFEB expression levels in TBHP-treated NP cells. Western blot results showed that apigenin treatment promoted the nuclear expression level of TFEB in a dose- and time-dependent manner (Figures 4A,B); an obvious decrease in nuclear TFEB expression was observed when NP cells were administrated with TBHP (Figures 4C,E), but apigenin treatment reversed the decreased TFEB nuclear transfer triggered by TBHP (Figures 4D,F), which was also confirmed by cell immunofluorescence analysis (Figures 4G,H), These data suggest that apigenin may promote the nuclear level of TFEB in TBHP-treated NP cells.

### Apigenin Alleviates TBHP-Induced Autophagy Flux Blockage Through TFEB in NP Cells

**FIGURE 5:**
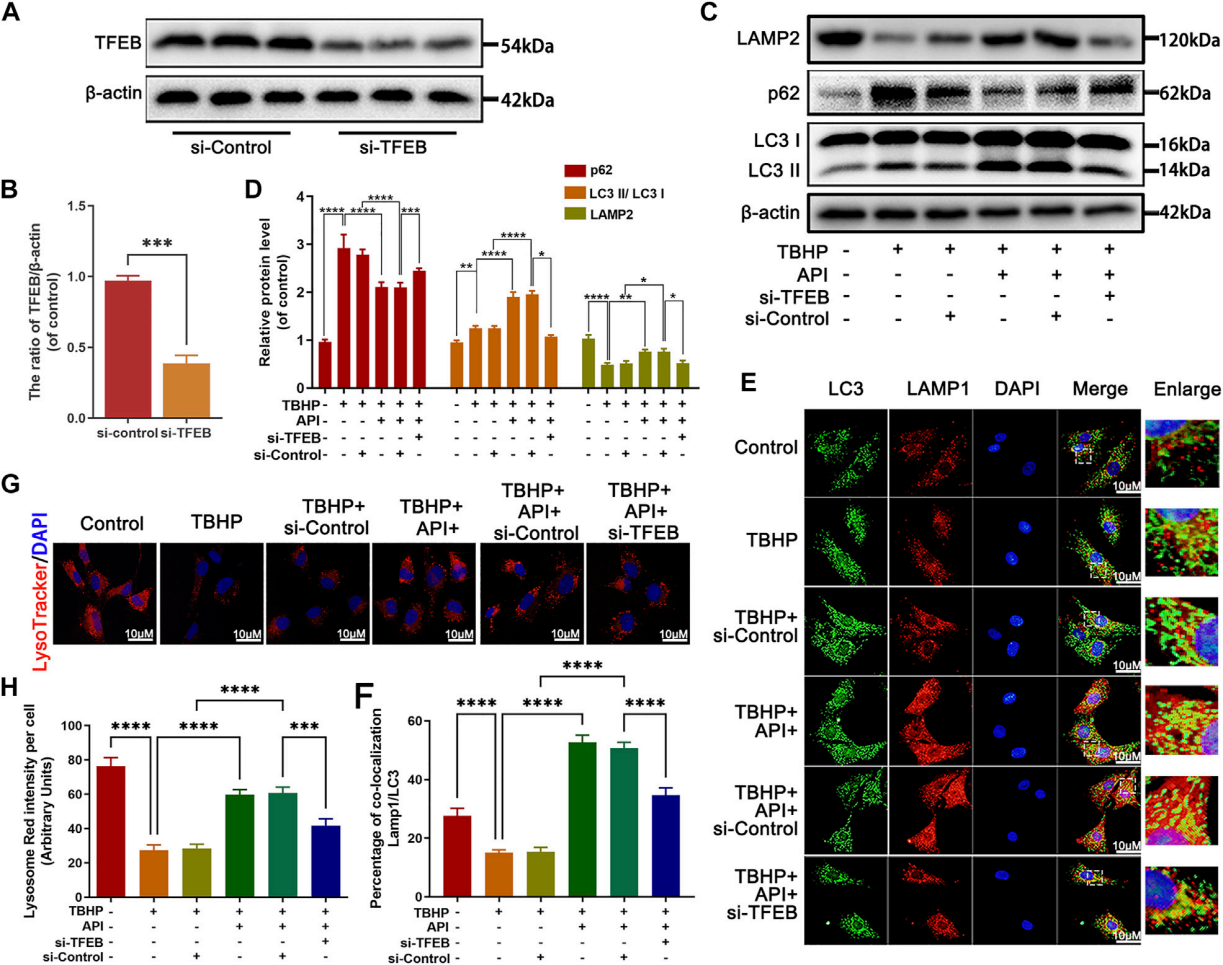
Apigenin (API) treatment rescues TBHP-induced autophagic flux blockage *via* TFEB in NP cells. The NP cells were pretreated with si-TFEB for 48 h followed with or without TBHP (50 μM) treatment and API (50 μM) treatment for 24 h. **(A,B)** The TFEB expression was measured by Western blot. **(C,D)** The expression of LAMP2, p62, and the ratio of LC3 II/LC3 I in TBHP- and API-treated NP cells were assessed by Western blot. **(E,F)** The double label immunofluorescence staining of LC3 and LAMP1 was performed in NP cells (scale bar: 10 μm). **(G,H)** The lysotracker staining in NP cells (scale bar: 10 μm). The data in the figures represent averages ±S.D. All data in the figures represent mean ± SD. ****p < 0.0001, ***p < 0.001, **p < 0.01, *p < 0.05.

It has been proved that TFEB regulated autophagic flux and lysosome function in NP cells; then, the function of apigenin on autophagic flux activity was evaluated after knocking down the TFEB expression by siRNA-TFEB (si-TFEB) in TBHP-treated NP cells. TFEB expression was successfully knocked down by si-TFEB in NP cells (Figures 5A,B). Meanwhile, as shown in Figures 5C,D, the effect of apigenin on improving the ratio of LC3-II/LC3-I and Lamp2 expression level as well as inhibiting p62 expression was remarkably reversed in the si-TFEB group compared to the siRNA-control (si-control) group. Besides, the immunofluorescence double staining intensity of LC3-II and Lamp1, which could represent the fusion of autophagy–lysosome, was enhanced by apigenin administration; however, this effect was eliminated by si-TFEB transfection (Figures 5E,F). Next, we also assessed the lysosomal enzyme activity by LysoTracker Red (LTR). As a specific lysosomotropic probe, it could be observed like a red fluorescence in fluorescence microscope, which changed with the lysosomal pH variation. As seen from Figures 5G,H, a weakened red fluorescence intensity was observed in NP cells under TBHP stimulation compared to the normal NP cells, indicating TBHP may lead to the impairment of lysosomal function, while apigenin treatment restored the low fluorescence intensity caused by TBHP stimulation. After transfecting si-TFEB, apigenin exhibited less effect on recovering the lysosome dysfunction induced by TBHP. All the aforementioned results indicate that apigenin alleviates TBHP-induced autophagy and lysosome dysfunction in NP cells.

### Apigenin Alleviates TBHP-Induced Apoptosis, Senescence and ECM Degradation in NP Cells *via* TFEB

**FIGURE 6:**
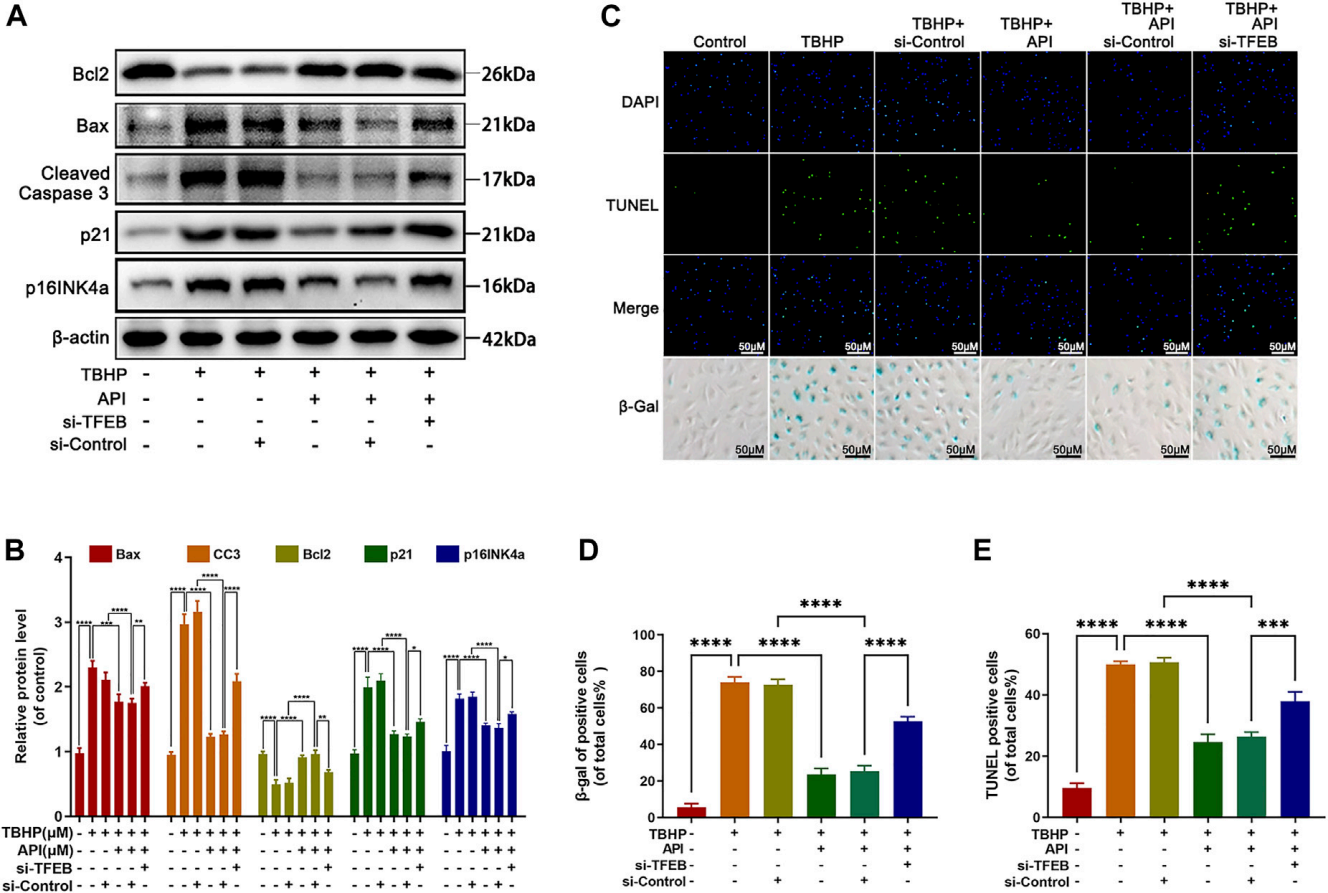
Apigenin (API) exhibits its protective effect against TBHP-induced apoptosis, senescence in NP cells *via* TFEB. NP cells were transfected with si-TFEB, then treated with or without TBHP (50 μM) and AP I (50 μM). **(A,B)** The expression of Bcl2, Bax, cleaved caspase 3, p21, and p16INK4a in NP cells were analyzed by Western blot. **(C–E)** TUNEL and SA-β-gel staining was conducted in NP cells treated as described above (scale bar: 50 μm). Data are represented as the mean ± SD. ****p < 0.0001, ***p < 0.001, **p < 0.01, *p < 0.05.

Then, we assessed whether apigenin exerted the antiapoptosis, anti-senescence, and anti-degradation of ECM ability *via* TFEB regulation in TBHP-treated NP cells. Apoptosis was detected by Western blot and TUNEL staining. As observed in Figures 6A,B, the Bcl2 expression level was increased, while Bax and cleaved caspase 3 expression levels were remarkably decreased by apigenin treatment. Interestingly, the deletion of TFEB abolished this phenomenon, which was also confirmed by TUNEL staining (Figures 6C,D). Besides, we detected the senescence-related indicators, p21 and p16INK4a expression levels, and the SA-β-gal activity in NP cells. Knocking down TFEB remarkably reversed the apigenin’s effect on reducing p21 and p16INK4a expression levels (Figures 6A,B), and the SA-β-gal–positive cells (Figures 6C,E). In the meantime, collagen II and aggrecan proteins levels were significantly increased, the MMP13 and ADAMTS5 proteins were remarkably decreased in TBHP-treated NP cells cocultured with apigenin and TFEB-control; while an opposite result was obtained when NP cells were cocultured with apigenin and si-TFEB (Supplementary Figures S4A,B). Furthermore, the increment of fluorescence intensity of collagen II was inhibited in the si-TFEB group; on the contrary, the MMP13 fluorescence intensity was enhanced (Supplementary Figures S4C,D). All data above suggest that apigenin may suppress senescence, apoptosis, and ECM degradation in NP cells caused by TBHP through regulating TFEB.

### Apigenin Activates AMPK/mTOR Pathway in NP Cells

As previous studies described, the AMPK/mTOR signaling cascade was known as one of the classic pathways regulating TFEB. Hence, we investigated whether this signal pathway was activated in NP cells under apigenin treatment. As shown in Supplementary Figures S5A–D, apigenin changed the p-AMPK and p-mTOR protein levels in a time- and dose-dependent manner; while no obvious variation was observed both in total AMPK and mTOR protein levels, indicating that apigenin activated the AMPK/mTOR pathway. Next, to detect the apigenin’s effect on TFEB whether through the AMPK/mTOR pathway, an AMPK inhibitor, compound C, was used in NP cells for inhibiting AMPK expression. As observed in Supplementary Figures S5E–J, compound C administration reversed the activation of the AMPK/mTOR pathway and TFEB nuclear translocation by apigenin. Besides, Western blot results also indicated compound C remarkably impaired apigenin-mediated enhancement of autophagy and autophagic flux in NP cells (Supplementary Figures S6A,B). In addition, the protection of apigenin against TBHP-induced apoptosis, senescence, and ECM degradation was also markedly attenuated by compound C (Supplementary Figures S6C–F). Hence, these results confirm that apigenin activated TFEB in NP cells through the AMPK-mTOR signaling pathway.

### Apigenin Treatment Ameliorates IVDD in Rats *In Vivo*

**FIGURE 7:**
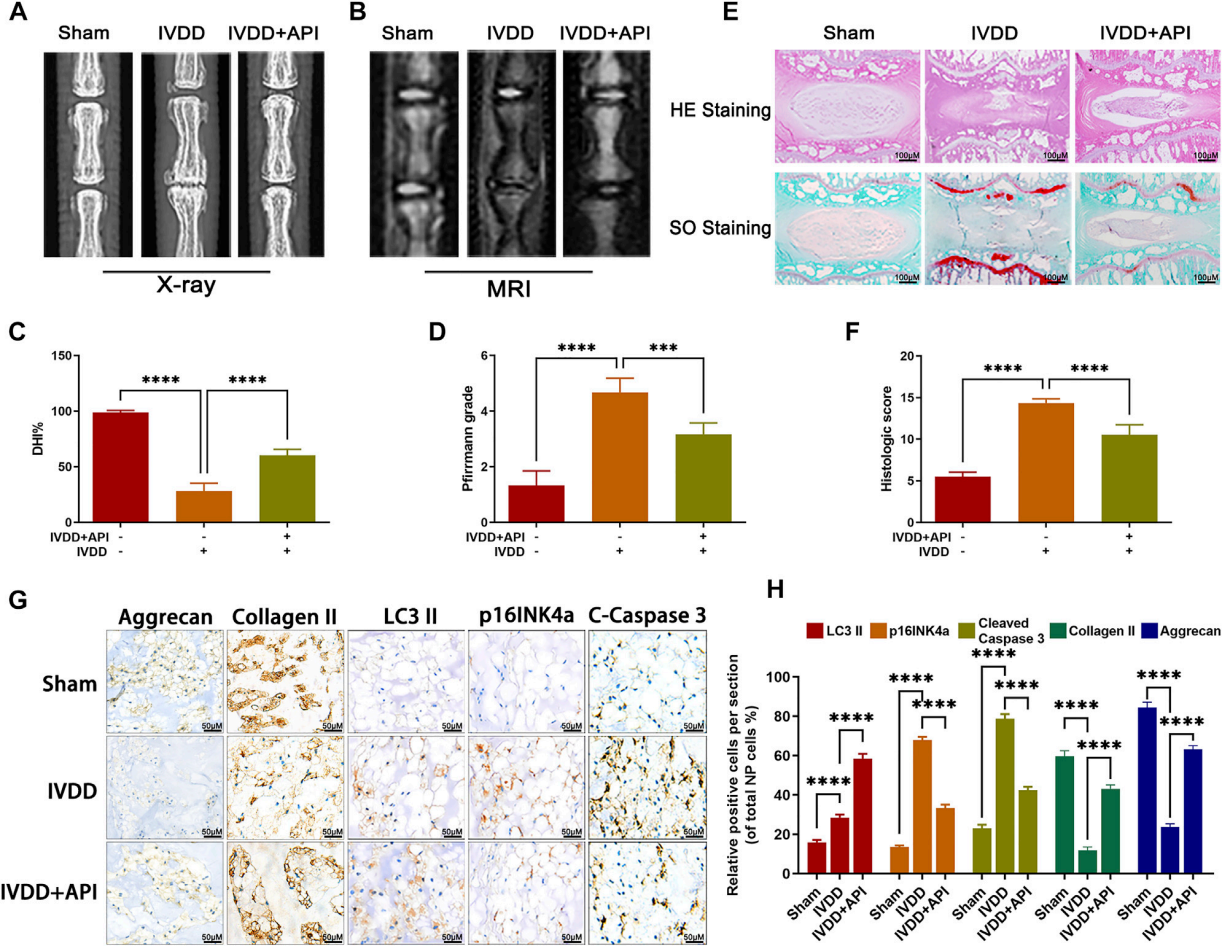
Apigenin (API) ameliorates rat IVDD progression *in vivo*. **(A,B)** Rat’s tails were taken X-ray and MRI imaging at 8 weeks after surgery. **(C,D)** The disc height index and Pfirrmann grade scores of rat tails disc were measured and analyzed at 8 weeks after surgery. **(E)** HE and SO staining of rat discs were also performed (scale bar: 100 μm). **(F)** The histological scores of the tails discs according to the histological grading scale. **(G,H)** The expression of LC3-II, p16INK4a, cleaved caspase 3 (C-Caspase 3), collagen II, and aggrecan in discs was evaluated by immunohistochemical staining (scale bar: 50 μm). The data in the figures represent mean ± S.D. Significant differences between groups are indicated as ****p < 0.0001.

In order to investigate the potential therapeutic effect of apigenin on IVDD *in vivo*, a needle puncture-induced IVDD model in rats was obtained. The X-ray and MRI images were obtained at 8 weeks after surgery. As observed in Figure 7B, the T2-weight signal intensity of the punctured section was gradually weakened at 8 weeks after surgery, but this phenomenon was delayed by apigenin treatment. The degeneration extent was further quantified by the Pfirrmann grading system (Figure 7D). Meanwhile, X-ray results showed that apigenin treatment alleviated the decline of disc height induced by puncture injury (Figures 7A,C). Apart from the aforementioned assessment, histomorphological changes between normal and damaged disc tissues with or without apigenin treatment were also assessed. HE staining and SO staining were performed to assess the variation of NP histological structure and proteoglycans and glycosaminoglycans, respectively. As observed in Figure 7E, the NP structure was gradually shriveled and NP cells were reduced and invaded by notable fibrous tissue in the IVDD group, while treatment with apigenin significantly delayed the disruption of IVD tissues, like more NP cells existed and less lamellar disorganization or fragmentation and ECM component containing in the disc. According to the analysis of the histological scores from SO staining (Figure 7F), apigenin administration clearly showed lower scores compared to the IVDD group, which further confirmed the protection of apigenin against IVDD progression. Besides, we detected the expression of LC3-II, p16INK4a, cleaved caspase 3, collagen II, and aggrecan by immunohistochemical staining, and the results were consistent with the Western blot results mentioned before (Figures 7G,H). Taken together, all these results suggest that apigenin participates in the therapy of IVDD and ameliorates its progression *in vivo*.

## Discussion

**FIGURE 8:**
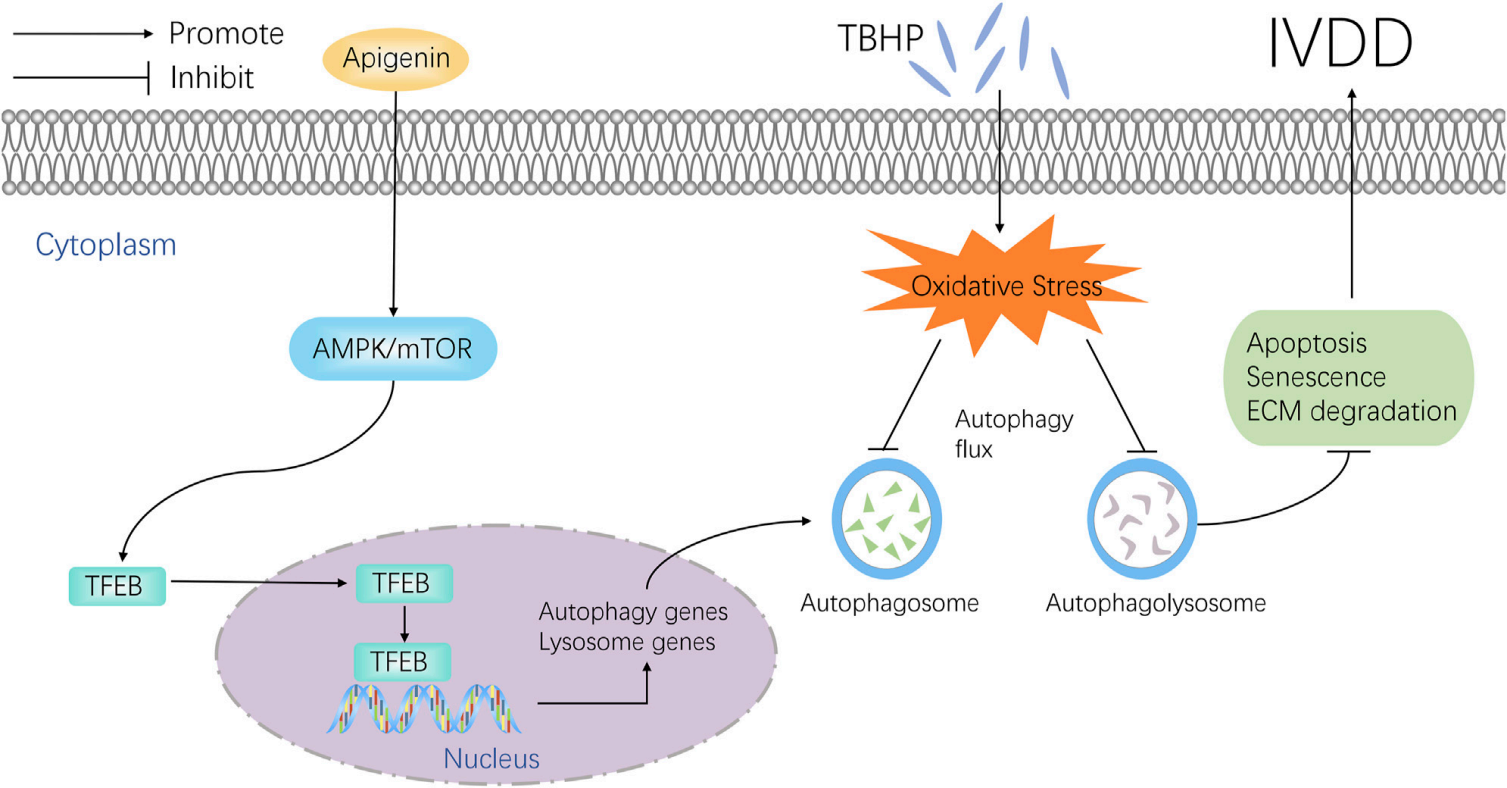
Potential molecular mechanism involved in apigenin treatment in NP cells.

IVDD is a clinical refractory degenerative disease, which has been reported as the major reason for low back pain (Lazary et al., 2014; Wilke et al., 2014). Current clinical medicine is only to alleviate the symptom, and most of them cannot reverse the pathology of IVDD. Thus, a drug focus on the pathological mechanisms to delay the IVDD progression is necessarily needed. This study found that apigenin can enhance autophagy and restore autophagic flux by regulating TFEB, which inhibits TBHP-induced apoptosis, senescence, and ECM anabolic and catabolism imbalance *in vitro*. The *in vivo* study also confirmed the effect of apigenin on IVDD (Figure 8).

The pathophysiological characteristics of aging-related degenerative diseases are referred to as multiple factors, such as inflammatory cytokines, mechanical load, and metabolic stress, leading to an increased level of reactive oxygen species (ROS) (Labuschagne and Brenkman, 2013; Ali et al., 2015). The overexpression of ROS seriously harms cell homeostasis and leads to the accumulation of damaged DNA and dysfunctional mitochondria, especially in degenerative diseases with weak antioxidant defense mechanisms (Grishko et al., 2009). Continuous release of ROS leads to oxidative stress, creating cell apoptosis and premature senescence, and ultimately leading to cell death (Lin et al., 2021). In this study, TBHP was selected as an ROS donor to create an oxidative stress condition *in vitro* study. As expected, in TBHP-treated NP cells, the antiapoptotic protein Bcl2 was significantly downregulated, while pro-apoptotic proteins Bax and cleaved caspase 3 were significantly upregulated. Meantime, the activity of SA-β-gel and senescence-related proteins p21 and p16INK4a expression levels were also increased in NP cells exposed to TBHP. ECM plays a vital role in maintaining the IVD physiological function, which contains two main components: collagen II and aggrecan that enhance the disc tensile strength and promote nutrient diffusion, respectively. Interestingly, TBHP-induced ECM synthesis/degradation imbalance was reversed by apigenin, as indicated by promoting collagen II and aggrecan expression, and inhibiting matrix degradation enzyme (MMP-13 and ADAMTS-5) expression, indicating that apigenin has a protective effect on NP cells against exposed to TBHP. In addition, our puncture-induced rat IVDD model further confirmed the results of the aforementioned *in vitro* experiments.

Autophagy, which means “self-eating,” maintains cell homeostasis by degrading and recovering damaged organelles and proteins in cells to cope with the increase in cell metabolism or external pressure. Previous studies have shown that normal NP cells accompanied with a low level of basal autophagy (Choi et al., 2016). In degenerated NP cells, autophagy activity also increases. In addition, other studies have also demonstrated that autophagy played an important role in the pathogenesis of IVDD (Zhang et al., 2016). For example, the research results of Yang Chao et al. showed that autophagy activation can alleviate mitochondrial dysfunction, cell apoptosis, and aging, which further delay IVDD progression (2018). Zheng Gang et al. pointed out that autophagy can inhibit the expression of pro-apoptotic proteins (cleaved caspase 3), and senescence-related protein (p16INK4a) in NP cells treated with TBHP and IVDD progression was accompanied with lysosomal activity impairment and decrease of autophagolysosome formation (Zheng et al., 2019a). Therefore, enhancing autophagic flux becomes a potential therapeutic strategy for IVDD. In our current work, we found that apigenin can activate autophagy-related indicator expression (as observed by the increased LC3-II expression in Supplementary Figure S1) and restore autophagy flux (as observed by the increased Lamp2 expression in Figures 5C,E) in TBHP-treated NP cells, indicating that apigenin alleviates the autophagic flux blockage caused by TBHP.

In order to clarify the action mechanism of apigenin, we focused on the upstream regulatory protein of autophagy activity, such as transcription factor (TFEB), the controller of autophagosome, and lysosome biogenesis. Normally, TFEB exists in the cytoplasm in the form of phosphorylation combining with the chaperone TFEB14-3-3 (Roczniak-Ferguson et al., 2012; Settembre et al., 2012). Once cell homeostasis is interrupted by internal or external signals (starvation, oxidative stress, metal toxicity), TFEB is dephosphorylated and dissociated from the complex, then the free dephosphorylated TFEB enters into the nucleus, participates in the regulation of autophagy and lysosomal biogenesis (Medina et al., 2015). This study showed that apigenin increased the expression of TFEB. Besides, the protective effect of apigenin against TBHP-induced apoptosis, senescence and ECM degradation were abolished by si-TFEB, indicating that apigenin exerts its protection ability on NP cells *via* regulating TFEB.

To find out the specific mechanism by which apigenin regulates TFEB, we focused on the AMPK pathway, a key metabolic sensor of regulating autophagy, which was activated by nutritional deficiency or increased AMP/ATP ratio (Shah et al., 2017). While in the cytoplasm, the increased phosphorylated AMPK suppressed mTOR activity, which promotes the dissociation of TFEB from the complex and transfers to the nucleus enhancing lysosomal and autophagy gene expression (Jiang et al., 2015; Wu et al., 2019; Zhou et al., 2019). In this study, we demonstrated that the AMPK/mTOR signaling pathway was activated by apigenin in a dose- and time-dependent manner in NP cells. In addition, an AMPK inhibitor, compound C, inhibited apigenin-mediated activation of the AMPK/mTOR signaling pathway and the effect on alleviating apoptosis, senescence, and ECM degradation in NP cells under oxidative stress. Together, our results confirmed that apigenin activated autophagy and regulated the TFEB level through the AMPK/mTOR signaling pathway. It is worth noting that the effect of apigenin on AMPK/mTOR signaling is consistent with previously reported studies (Lu et al., 2019; Zhang et al., 2020a).

In summary, the current study demonstrated that apigenin exerts a protective effect against TBHP-induced apoptosis, senescence, and ECM degradation in NP cells *via* restoration of autophagic flux targeting the TFEB level *via* the AMPK-mTOR signaling pathway. In addition, *in vivo* experiments confirmed the therapeutic effect of apigenin may delay IVDD in rats. These findings provide new insights into understanding the potential of Apigenin in the treatment of IVDD.

## Data Availability

The raw data supporting the conclusion of this article will be made available by the authors, without undue reservation.
