# Supplementary material for: Apigenin Alleviates Intervertebral Disc Degeneration via Restoring Autophagy Flux in Nucleus Pulposus Cells
Source: Front Cell Dev Biol. 2022 Jan 14;9:787278. doi: 10.3389/fcell.2021.787278 (PMC8795835; doi:10.3389/fcell.2021.787278)
Supplement: Supplementary file 1 [file DataSheet1.PDF]

Figure S1

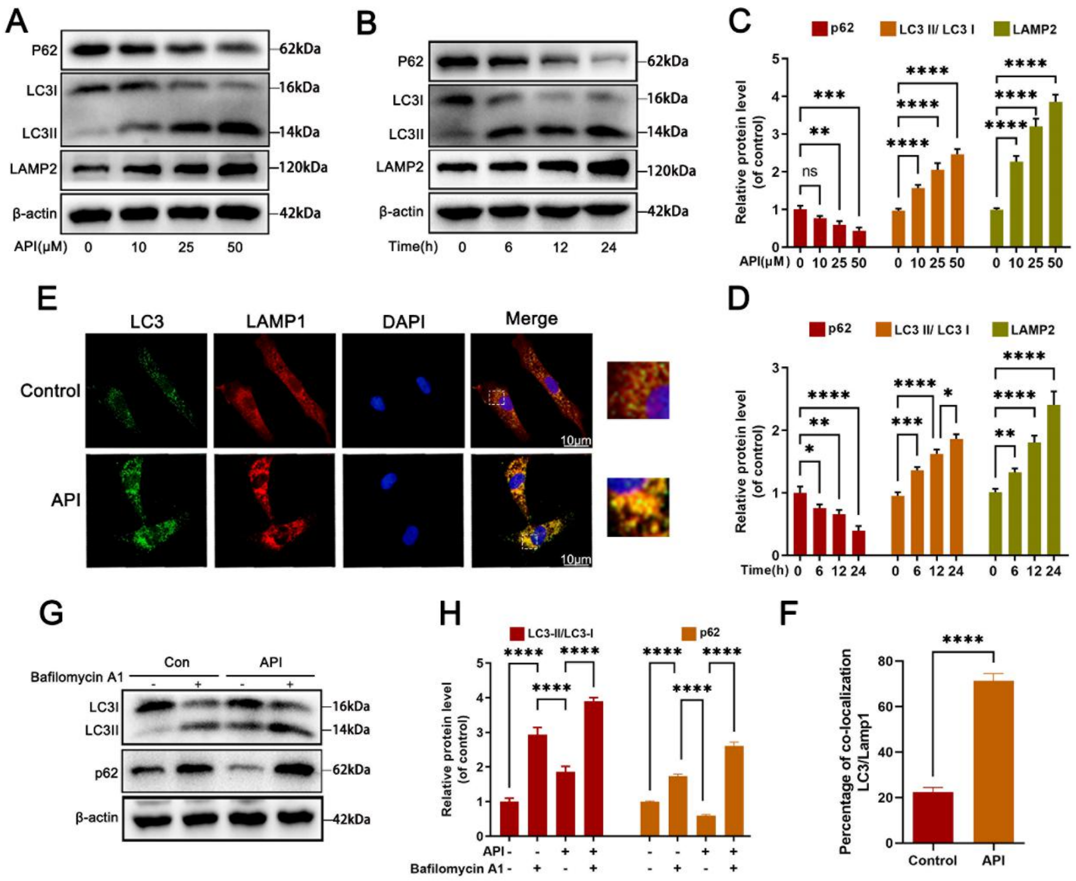

Figure S1. Apigenin (API) treatment activates autophagy and enhances autophagic flux in NP cells. **(A-B)** The expression of autophagy-related protein, including p62, LC3II/LC3I and LAMP2 in NP cells, which treated with different concentrations of API for 24h or treated with API (50μM) for different time, were evaluated by western blot. **(C-D)** Quantification of p62, LC3II/LC3I and LAMP2 by immunoblot. **(E)** Immunofluorescence double staining of LC3 and LAMP1 was performed in NP cells (scale bar: 10μm). **(F)** The fluorescence intensity of LC3 and LAMP1 in NP cells was quantified. **(G-H)** The protein expression of LC3 and p62 in NP cells. Data are represented as the mean ± SD. ****P < 0.0001, ***P <0.001, **P < 0.01, *P < 0.05.

Figure S2

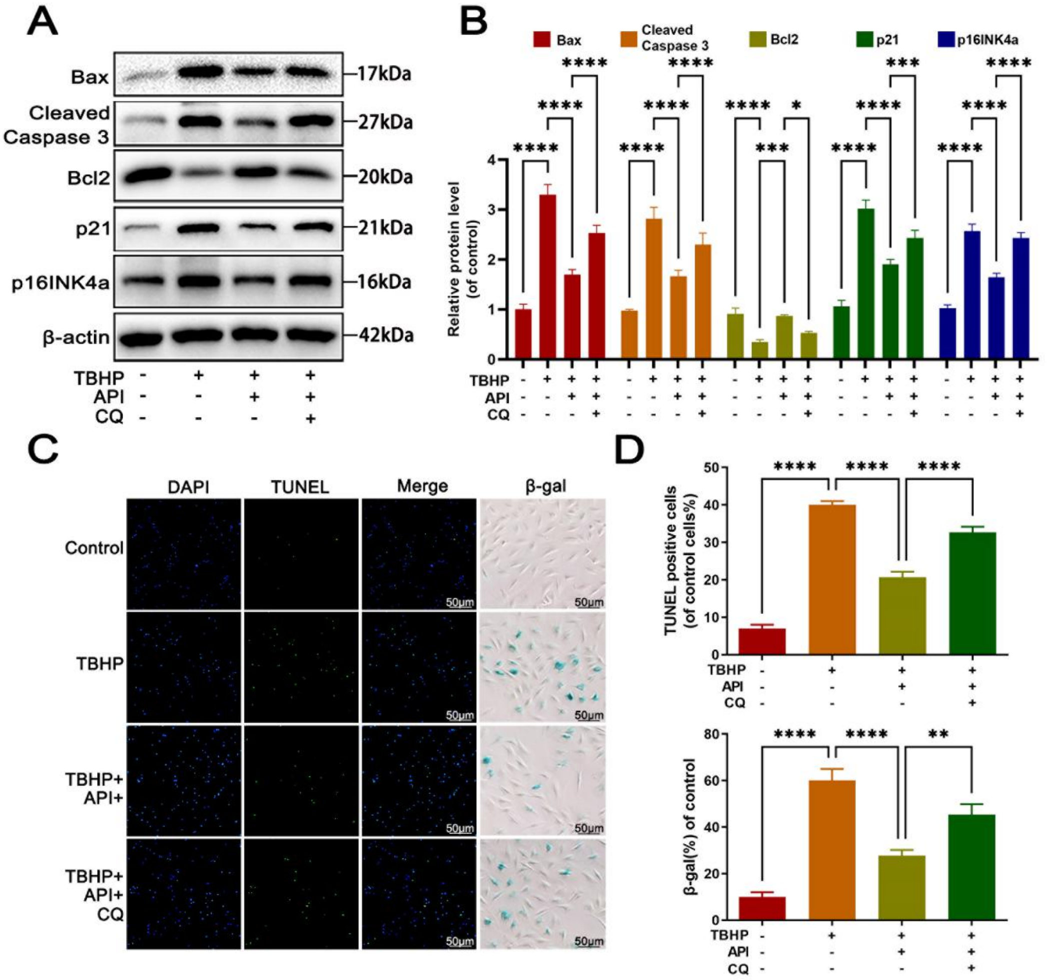

Figure S2. CQ reverses the protective effects of Apigenin against apoptosis and senescence in NP cells exposed to TBHP. NP cells were treated with CQ for 6h before TBHP (50μM) and API (50μM) administration. **(A-B)** The protein expression of Bcl2, Bax, Cleaved Caspase 3, p21 and p16INK4a in NP cells. **(C-D)** TUNEL and SA-β-gel staining assay were performed in NP cells as treated above (scale bar: 50μm). Data are represented as the mean ± SD. ****P < 0.0001, ***P <0.001, **P < 0.01, *P < 0.05.

Figure S3

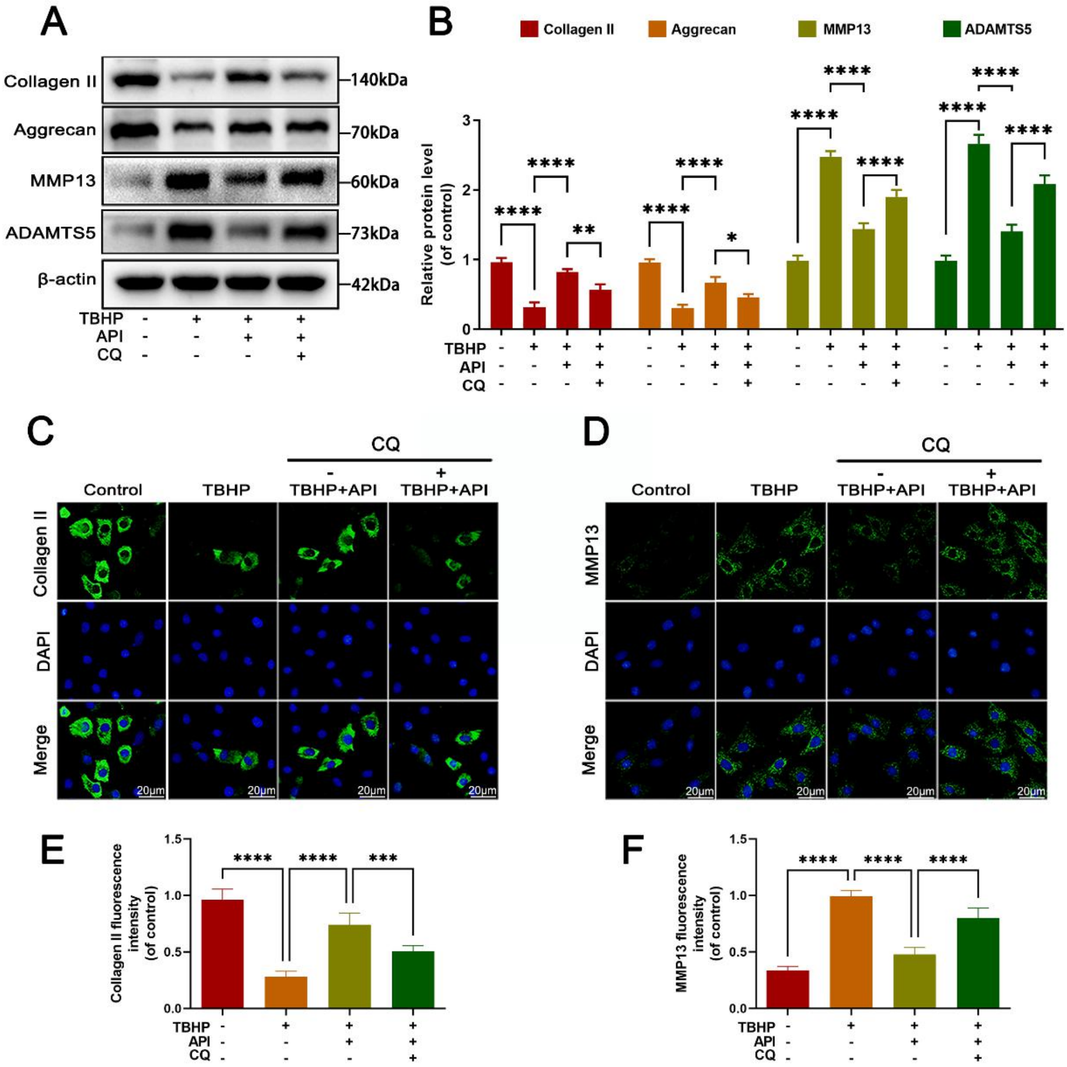

Figure S3. CQ reverses the protective effects of Apigenin against ECM degradation in NP cells exposed to TBHP (50μM). **(A-B)** The protein expression of Collagen II, Aggrecan, MMP13 and ADAMTS5 in NP cells. **(C-D)** Representative image of immunofluorescence staining of Collagen II and MMP13 in NP cells (scale bar: 20μm). **(E-F)** The fluorescence intensity of Collagen II and MMP13 was quantified by Image J. Data are represented as the mean ± SD. ****P < 0.0001, ***P <0.001, **P < 0.01.

Figure S4

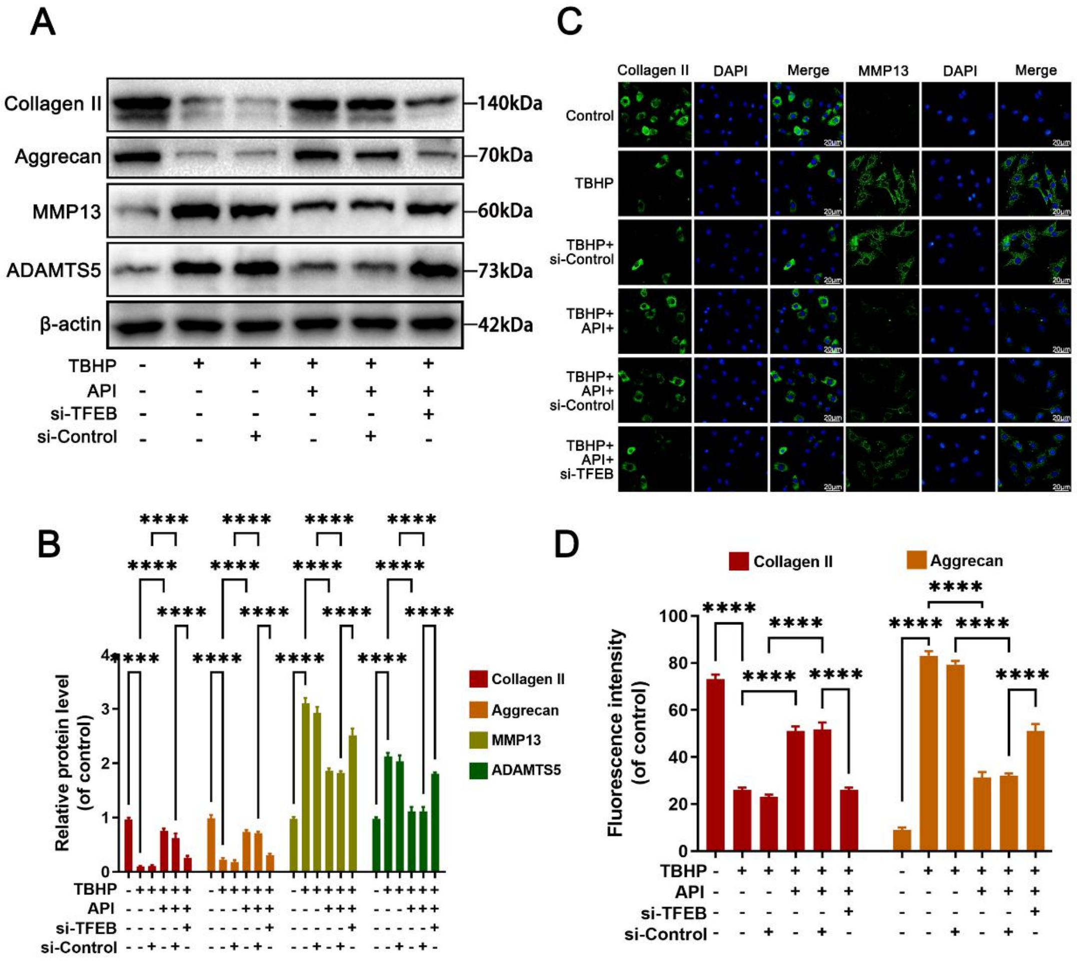

Figure S4. Apigenin (API) alleviates TBHP-induced ECM degradation in NP cells *via* TFEB. NP cells were transfected with si-TFEB, then treated with or without TBHP (50μM) and API (50μM). **(A)** The expression of Collagen II, Aggrecan, MMP13 and ADAMTS5 in NP cells. **(B)** Quantification of Collagen II, Aggrecan, MMP13 and ADAMTS5 by immunoblot. **(C)** The cell immunofluorescence images of Collagen II and MMP13(scale bar: 20μm). **(D)** Quantitative analysis of the fluorescence intensity of Collagen II and MMP13 was done by Image J. Data are represented as the mean ± SD. ****P < 0.0001, ***P <0.001, **P < 0.01, *P < 0.05.

Figure S5

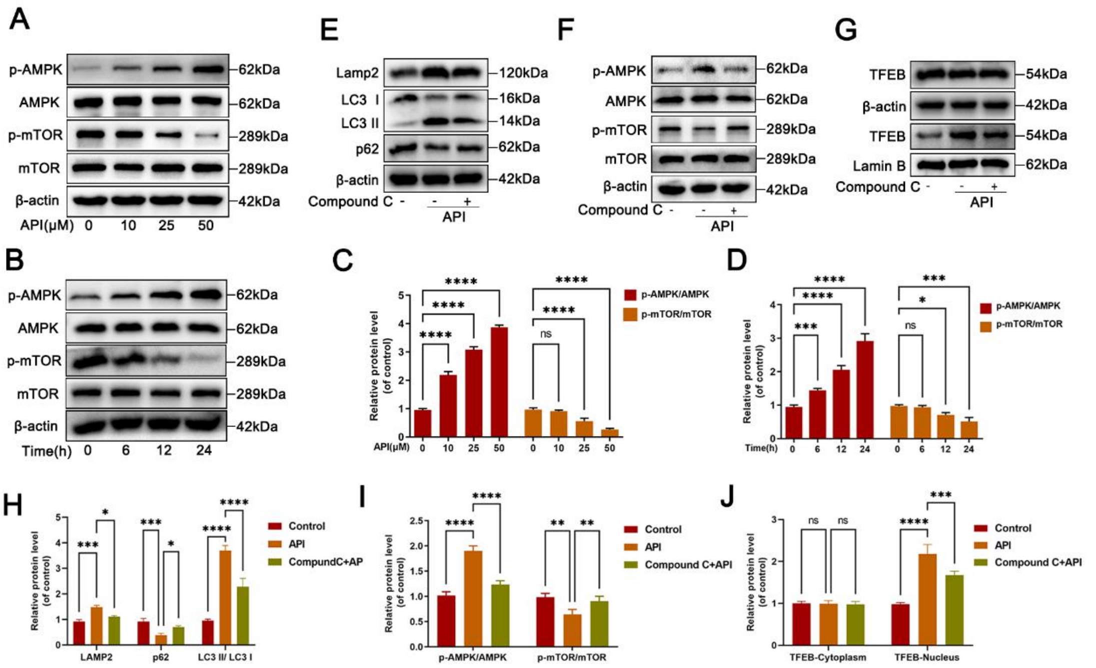

Figure S5. AMPK/mTOR pathway is involved in Apigenin (API)-induced activation of autophagy in NP cells. NP cells treated with API for different concentration and time, or NP cells treated with compound C (5μM) and API (50μM) for 24h. **(A-D)** Western blot results of expression level of AMPK, p-AMPK, mTOR and p-mTOR in NP cells. **(E-J)** The levels of LAMP2, LC3II/LC3I, p62, AMPK, p-AMPK, mTOR, p-mTOR and TFEB proteins in NP cells treated as described. Data are represented as the mean ± SD. ****P < 0.0001, ***P <0.001, **P < 0.01, *P < 0.05.

Figure S6

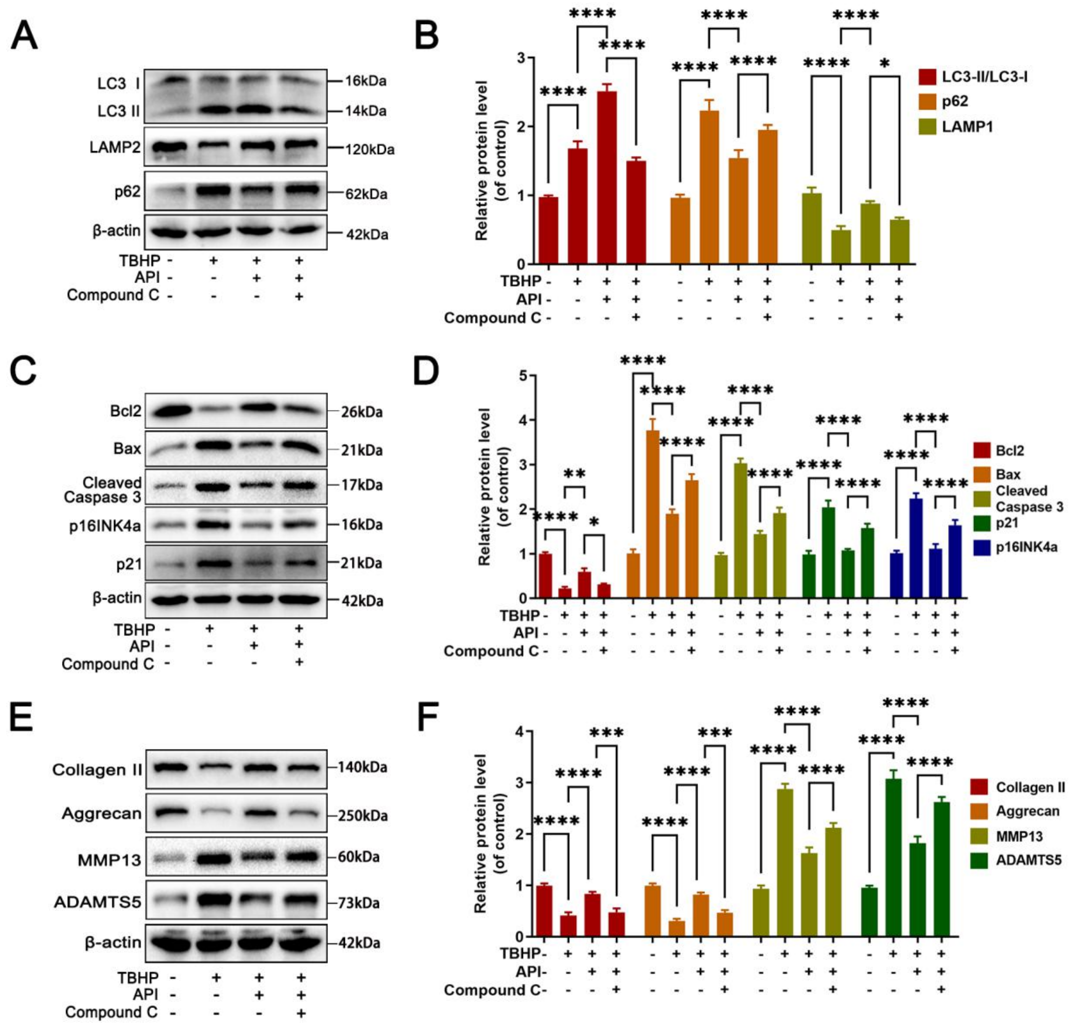

Figure S6. Compound C inhibits the protective effect of Apigenin (API) in TBHP-exposed NP cells. NP cells were treated with TBHP (50μM), API (50μM) and compound C (5μM) for 24h. **(A, C, E)** The protein expression of LC3, LAMP2, p62, Bcl2, Bax, Cleaved Caspase 3, p21, p16INK4a, Collagen II, Aggrecan, MMP13 and ADAMTS5 in NP cells as treated above were measured by western blot. **(B, D, F)** Quantitative analysis of above protein were measured by Image J. Data are represented as the mean ± SD. ****P < 0.0001, ***P <0.001, *P < 0.05.
